# Technical note and first results on JET 7 thromboaspiration device for T-ICA occlusions

**DOI:** 10.1186/s12883-022-02784-1

**Published:** 2022-07-12

**Authors:** Daniele Giuseppe Romano, Giulia Frauenfelder, Francesco Diana, Renato Saponiero

**Affiliations:** 1 Department of Neuroradiology, A.O.U. San Giovanni di Dio e Ruggi d’Aragona, Via San Leonardo 1, 84100 Salerno, Italy; 2 Department of Neuroradiology, A.O.U. San Giovanni di Dio e Ruggi d’Aragona, Via San Leonardo 1, Salerno, Italy; 3 Department of Neuroradiology, A.O.U. San Giovanni di Dio e Ruggi d’Aragona, Via San Leonardo 1, Salerno, Italy; 4 Department of Neuroradiology, A.O.U. San Giovanni di Dio e Ruggi d’Aragona, Via San Leonardo 1, Salerno, Italy

**Keywords:** Stroke, Thrombectomy, Angiography, Technique, Catheter

## Abstract

**Background:**

To describe technical features and initial results of a novel large-bore reperfusion catheter as first thromboaspiration approach for endovascular stroke treatment in terminal internal carotid artery (T-ICA) occlusions.

**Methods:**

All patients treated with A Direct Aspiration first-Pass Technique (ADAPT) using JET 7 “Standard Tip” Penumbra Reperfusion catheter for acute T-ICA occlusion were retrospectively included in the study. Baseline data, puncture to recanalization time, number of attempts, switch to second device/technique rate and successful recanalization rate were assessed. Successful recanalization was defined by a thrombolysis in cerebral infarction (TICI) score ≥ 2b and favorable functional outcome was defined according to modified Rankin scale (score, 0–2). Catheter specifics and thromboaspiration reperfusion technique with JET 7 were reported.

**Results:**

A total of 21 patients who underwent ADAPT with JET 7 Reperfusion catheter were enrolled for the final analysis. ADAPT was performed as first approach in all cases (100%). First attempt successful recanalization (eTICI ≥2b) was obtained in 90,5% of cases. Mean puncture to recanalization time was 16 minutes. Final successful recanalization was reached in 96.5%. Functional independence at 90 was achieved in 57,1% cases. Symptomatic intracranial hemorrhage occurred in one patient within 24 h.

**Conclusion:**

The large-bore JET 7 reperfusion catheter could be considered as first-line in patients with acute T-ICA occlusion, allowing rapid recanalization and low rate of rescue therapy with stent retriver. Further series and/or trial evaluation are required to confirm our results.

## Background

The necessity for rapid recanalization in acute ischemic stroke (AIS) to restore flow through the occluded vessel is well established [1]. Newer and more performant large-bore aspiration catheters have recently emerged as a valid therapeutic option in treating patients with AIS. A recent RCT [2] according to European Stroke Organization guidelines [3], supports the use of “A Direct Aspiration first-Pass Technique” (ADAPT) as alternative to stent retriever in first-line thrombectomy. Suction force and tip diameter are the most crucial factors for successful mechanical thromboaspiration with ADAPT [4], as with a larger tip diameter, the aspiration force would increase. This concept needs to be verified in patients with extensive clot burden such as Carotid Terminal Occlusion (CTO) and Non-T Occlusion (CNTO) [5], as still poor outcome is reported in these cases because of slower recanalization, higher complication rate and non-target embolization [6]. As never reported before, our aim was to assess preliminary experience about technical and clinical outcomes of a novel large-bore reperfusion catheter (Penumbra JET™ 7 Reperfusion Catheter *Standard Tip*) in exclusive Terminal-ICA(T-ICA) acute occlusions.

## Methods

### Patient’s selection

Over a period of 6 months, a retrospective analysis of prospectively collected data was conducted at our Institution, from all patients whose mechanical thrombectomy interventions for T-ICA occlusions involved JET 7 catheter. Inclusion criteria were: age 18 ≥ years; time window within 6 h; National Institutes of Health Stroke Scale (NIHSS) score ≥ 6 and modified Rankin Scale (mRS) score of 0-2 at baseline; ASPECT ≥6 at baseline; anterior circulation stroke with CTO; no intracerebral hemorrhage (ICH. A written informed consent for the procedure was obtained from patients or from an approved delegate in case of unconscious patient.

### Technical note

The Penumbra JET™ 7 “Standard Tip” (Penumbra, Inc. USA One Penumbra Place Alameda, CA, USA) is a large-bore extra-flexible reperfusion catheter, intended for use in the revascularization of patients with AIS secondary to intracranial large vessel occlusions. It has been launched from Penumbra in European states since December 2019. A recall from U.S. Food and Drug Administration (FDA) was applied to the Penumbra JET 7 Reperfusion Catheter *Xtra Flex Technology* (JET 7 Xtra Flex) because of serious injury while used for removing clots in stroke patients; the Penumbra JET 7 “Standard Tip”, analyzed in our study, isn’t affected by the recall and it is routinely used worldwide. This catheter is designed to perform direct aspiration of the clot and to provide stable support in coaxial or triaxial thrombectomy approach. It is characterized with 20 transitions to support trackability and navigation and with distal coil wind for flexibility. It has a rigid proximal part and a flexible distal part, allowing for easy navigability and stable support. Technical specifics, compared with previous generation of similar large bore catheter, ACE68, are reported in Table 1.

**Table 1:** Technical specific for JET 7 in comparison with the previous ACE 68, from Penumbra Reperfusion device family

Dimension	ACE™ 68	JET 7
Proximal OD	0.084 in Max	0.085 in Max
Proximal ID	0.068 in Min	0.072 in Min
Distal OD	0.084 in Max	0.085 in Max
Distal ID	0.068 in Min	0.072 in Min
Effective length	115, 120, 125, 127, 132 cm	Same
Distal flex length	30 cm	Same
Coating length	30 cm	Same
**Indication**	Penumbra Reperfusion Catheters and Separators As part of the Penumbra System, the Reperfusion Catheters and Separators are indicated for revascularization of patients with AIS secondary to intracranial large vessel occlusive disease (within the internal carotid, middle cerebral – M1 and M2 segments, basilar, and vertebral arteries) within 8 hours of symptom onset. Patients who are ineligible for intravenous tissue plasminogen activator (IV t-PA) or who fail IV t-PA therapy are candidates for treatment.	Same

### Endovascular technique (EVT)

EVTs were performed by experienced neurointerventionalists. A large-bore 088 Neuronmax guide catheter (Penumbra Inc., CA, USA) was advanced as distal as possible in ICA, over a 5/6F 125-130 cm diagnostic catheter. Thereafter, the JET 7 was advanced to contact the thrombus over a Velocity or 3MAX delivery microcatheters (Penumbra, Alameda, CA, USA) and a Synchro 0.014 microwire (Stryker, Fremont, CA, USA). Guide catheter was further advanced, at least beyond petrous tract of ICA. Microcatheter and microwire were then removed; continuous negative aspiration was connected to JET 7 using a Penumbra pump System and the aspiration catheter was slightly further advanced to ensure firm engagement of the thrombus. Simultaneously, manual aspiration through a 60 cc VacLok® Vacuum Pressure Syringe was performed from guide cathater. After at least 90 seconds of continuous aspiration, the reperfusion catheter was gradually removed. When ADAPT revascularization was not achieved after three attemps, the first operator would proceed with a stent retriever in combination with aspiration (Solumbra technique).

### Outcome measurements

Primary outcome was successful reperfusion grade, as “extended thrombolysis in cerebral infarction” (eTICI) ≥ 2b. Secondary outcomes were: time from groin puncture to reperfusion, first attempt recanalization (FAR), embolism of new territories, symptomatic intracranial hemorrhage within 24 h, mRS and mortality within 90-day follow-up. Descriptive statistics included the number of observations, mean and SD, and median for continuous variables. Categorical variables were expressed as frequencies (percentages).

## Results

**Fig. 1:**
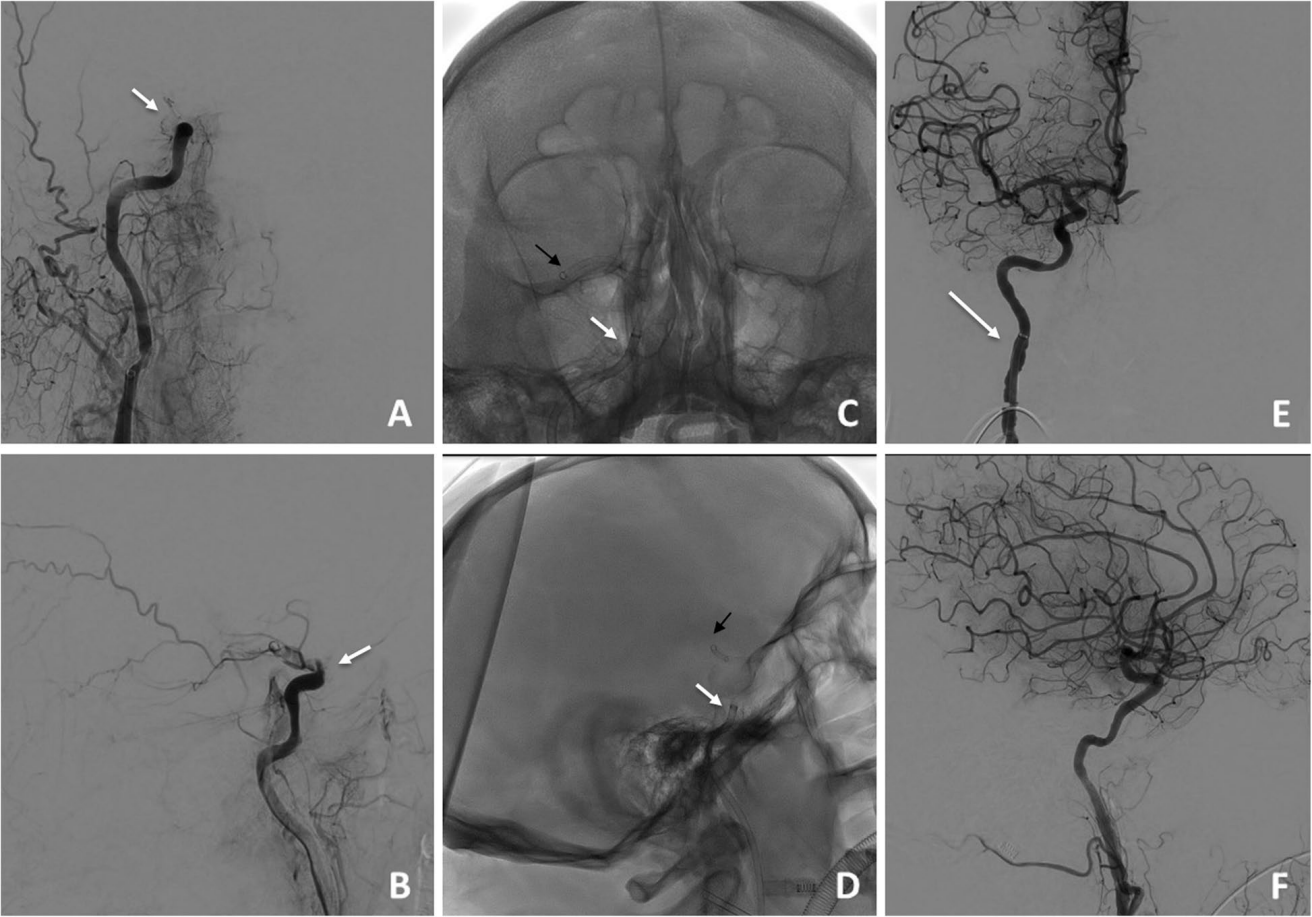
Acute right T-ICA occlusion at ophthalmic tract (with arrow in *a* and *b*). Neuronmax 088 guide catheter is advanced as distal as possible in the proximal cavernous tract of right ICA (white arrow in *c* and *d*), and tip of JET 7 thromboaspiration device is advanced to contact and incorporate the long clot, reaching the middle cerebral artery (black arrow in *c* and *d*). After one attempt with ADAPT technique, eTICI 3 was obtained. Please note Neuronmax-induced mechanical vasospasm and reduction of ICA caliber after recanalization (white arrow in *e*)

From December 2019 to June 2020, a total of 83 patients were treated with mechanical thrombectomy for AIS of large vessel occlusion (LVO) at our Institution; ADAPT with JET 7 for T-ICA occlusion was performed in a total of 21 patients. Demographic and clinical data are reported in Table 2. Stroke etiology was cardioembolic in 55%, dissection in 30%, hypercoagulability in 10%, and atherosclerotic in 5% of cases. Results are summarized in Table 3. JET 7 with ADAPT obtained technical success (eTICI ≥2b) in 90,5%. The distal catheter tip was in contact with the clot in 90,5% cases. At 90 days mRS 0-2 days was obtained in 52,4% (Fig. 1). When JET 7 did not obtain successful recanalization after 3 attemps, a stent retriever was incorporated as rescue in 1 case (eTICI = 2b). Mortality rate at 90 days was 14,3% (*n* = 3/21).

**Table 2:** Demographic and clinical baseline data

Patients data	Results (*n =* 21) [SD]
Age	69,2 [48-83]
Men	9 (42,8%)
Baseline NIHSS	13 [5-22]
Baseline ASPECT	8 [6-10]
Onset to door (min)	135 [63-350]
Hypertension	10 (47,6%)
Diabetes	5 (23,8%)
Hyperlipidemia	3 (14,3%)
Smoking	8 (38,1%)
Atrial Fibrillation	9 (42,8%)
Previous Stroke	2 (9,5%)

(*NIHSS* National Institutes of Health Stroke Scale, *ASPECT* Alberta Stroke Program Early CT score)

**Table 3:** Technical and clinical outcomes of JET 7 thromboaspiration device in T-ICA occlusions

Outcome measuremets	Results (*n =* 21) [SD]
Onset to puncture time (min)	179 [85-395]
Puncture to recanalization time (min)	16 [9-35]
eTICI ≥2b	19/21 (90,5%)
FAR	17/21 (80,9%)
Switch to other devices	1/21 (4,7%)
Symptomatic hemorrage	1/21 (4,7%)
DE	1/21 (4,7%)
Discharge NIHSS	6 [0-18]
mRS ≤ 2 at 90 days	12/21 (57,1%)

(*NIHSS*: National Institutes of Health Stroke Scale, *TICI* Thrombolysis In Cerebral Infarction, *FAR* First Attempt Recanalization, DE Distal embolization, *mRS* Modified Rankin Scale, *PTR* Puncture to Recanalization)

## Discussion

About endovascular treatment of T-ICA, the most recent literature emphasizes the importance of combining stent retriever with proximal thromboaspiration to obtain higher rates of first-pass reperfusion, correlated with technical and clinical success. The SAVE study [7] demonstrated significant higher rates of successful reperfusion (mTICI≥2b) in Stent-retriever Assisted Vacuum-locked Extraction (SAVE) group compared to ADAPT alone (93.5% vs 75.0%; *p* = 0.006). Previous reported data, demonstrated that CTOs are independent predictors for worse angiographic outcomes in ADAPT [8]. Possible explanations for this observation are that CTO have higher thrombus burden and not optimal ratios of vessel size to aspiration-catheter diameter. In our study, EVT using JET7 showed a short procedural time; low rate of switch to second-line stent retriever has been already reported [9]. These results are presumed to be related to the larger tip in order to aspirate ICA thrombus and, despite its size, superior trackability compared with prior versions [10]. Our standardized technique was based on the use of a proximal largebore guide-catheter, providing both intracranial support for navigation also in sever tortuosity and proximal anterograde flow control/arrest due to catheter-induced ICA mechanical vasospasm. The adopted technique allowed us to achieve rapid and efficacy recanalization in CTO, with TICI≥2b in 90,5% of cases. Additionally, successful recanalization rate was comparable to that of previous studies using stent-retrievers [11]. The use of JET 7 has been demonstrated to be safe and effective in different thrombectomy techniques and in other occlusion site (M1, M2, P1 segments) with mTICI 2b/3 achieved in 86% [12, 13]. Limitations were: a retrospective, non-consecutive design; limited number of cases; lack of a first-line stent retriever control group.

## Conclusion

JET7 reperfusion catheter may be considered a first-line choice for thromboaspiration in patients with acute T-ICA occlusion. More prospective studies are needed to confirm out results.

## Data Availability

The datasets used and/or analysed during the current study are available from the corresponding author on reasonable request.

## Abbreviations

T-ICA: Terminal internal carotid artery
ADAPT: A Direct Aspiration first-Pass Technique
eTICI: Extended thrombolysis in cerebral infarction
AIS: Acute ischemic stroke
CTO: Carotid Terminal Occlusion
CNTO: Carotid Non-T Occlusion
NIHSS: National Institutes of Health Stroke Scale
mRS: Modified Rankin Scale
EVT: Endovascular Technique
FAR: first attempt recanalization
SAVE: Stent-retriever Assisted Vacuum-locked Extraction
DE: Distal embolization
